# Sarcomere length of the vastus intermedius with the knee joint angle change

**DOI:** 10.14814/phy2.14771

**Published:** 2021-03-02

**Authors:** Ryosuke Ando, Keigo Taniguchi, Shin Kikuchi, Shogo Mizoguchi, Mineko Fujimiya, Masaki Katayose, Hiroshi Akima

**Affiliations:** ^1^ Department of Sports Research Japan Institute of Sports Sciences (JISS) Tokyo Japan; ^2^ School of Health Sciences Sapporo Medical University Sapporo Japan; ^3^ School of Medicine Sapporo Medical University Sapporo Japan; ^4^ Research Center of Health, Physical Fitness and Sports Nagoya University Nagoya Japan

**Keywords:** cadaver, electron microscope, force–length relation, quadriceps femoris

## Abstract

The force–length relation of the skeletal muscles is an important factor influencing the joint torque at a given joint angle. We aimed to clarify the relationship between the resting sarcomere length and knee joint angle in the vastus intermedius (VI) and to compare it with that of the vastus lateralis (VL). The left and right legs were fixed at knee joint angles of 0° and 90°, respectively, in seven cadavers (age at the time of death: 70–91 years). Muscle tissues were dissected by necropsy of the VL and the VI, and electron microscopy images were obtained to calculate the sarcomere length. At knee joint angles of 0° and 90°, the VL sarcomere length was 2.28 ± 0.49 μm and 2.30 ± 0.48 μm, respectively, and the VI sarcomere length was 2.19 ± 0.35 μm and 2.46 ± 0.53 μm, respectively, with a significant difference between the two (*p* = 0.028). The magnitude of sarcomere length changes with knee joint angle changes was significantly greater for the VI (0.27 ± 0.20 μm) than for the VL (0.02 ± 0.09 μm) (*p* = 0.009). Thus, knee joint angle changes may affect the passive and active tension produced by the VI more than those produced by the VL.

## Graphical abstract

The sarcomere length of the vastus lateralis was not significantly different between joint angles, whereas that of the vastus intermedius was increased at the flexed knee joint angle compared to the extended knee joint angle.

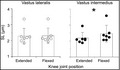

## 1 INTRODUCTION

The force–length relation of the skeletal muscle is a major factor affecting the joint torque at a given joint angle (Gordon et al., 1966). Two studies have estimated the force–length curve of the quadriceps femoris with knee joint angle changes (Cutts, 1988; Herzog et al., 1990). With respect to the vastus lateralis (VL), both studies found that with knee joint angle changes, the VL active sarcomere length (SL) changes over the ascending limb, plateau region, and descending limb of the force–length curve (e.g., 2.17–3.53 μm; Cutts, 1988). In contrast, Cutts (1988) found that the vastus intermedius (VI) active SL changes only in the ascending limb of the curve (1.97–2.48 μm), whereas Herzog et al. (1990) found that it changes from ascending to descending limbs, as in the case of the VL. This discrepancy might be due to small sample sizes (a number of cadavers were three and four in the studies of Cutts (1998) and Herzog et al. (1990), respectively) and the estimation of the resting SL at a flexed knee joint angle. Both studies directly measured the resting SL at the extended knee joint angle (i.e., anatomical position) in cadavers and measured the muscle length with knee joint angle changes using radiographs in living young men. Thus, they estimated the resting SL at flexed knee joint angles by combined cadaveric and in vivo measurements. Unfortunately, it is difficult to assess SL with joint angle changes by direct measurement in cadavers.

To overcome this problem, direct in vivo microendoscopy measurements have been performed in other muscles (Chen & Delp, 2016; Cromie et al., 2013; Llewellyn et al., 2008). Cromie et al. (2013) reported changes in the SL of the extensor carpi radialis brevis with wrist extension and flexion. Therefore, the resting VI SL should be directly measured at various knee joint angles in vivo using microendoscopy to clarify the discrepancy in the results between the two previous studies (Cutts, 1988; Herzog et al., 1990). However, a concern is that microendoscopy cannot be performed for the VI in vivo because it is a deep muscle that is almost completely covered by the superficial quadriceps components (Watanabe & Akima, 2009).

Joint flexibility is quite low in formalin fixed cadavers; therefore, SL with joint angle changes cannot be examined. However, joint flexibility is dramatically greater in cadavers fixed by Thiel's method (Thiel, 2002) compared to those fixed in formalin. We were not able to obtain electron microscopy images with clear sarcomere arrays in samples from cadavers in our preliminary experiment, yet this has been demonstrated in a previous study (Benkhadra et al., 2011). Thus, in the present study, we fixed the knee joint at a flexed knee joint angle (90º) in cadavers before formalin fixation. This study aimed to clarify the relationship between the resting SL and knee joint angle in the VI and to compare it with that of the VL.

## 2 MATERIALS & METHODS

### 2.1 Ethical approval

The experimental protocols were approved by the Ethics Committee of the School of Medicine, Sapporo Medical University, and the Research Center of Health, Physical Fitness and Sports, Nagoya University.

### 2.2 Cadavers

**FIGURE 1:**
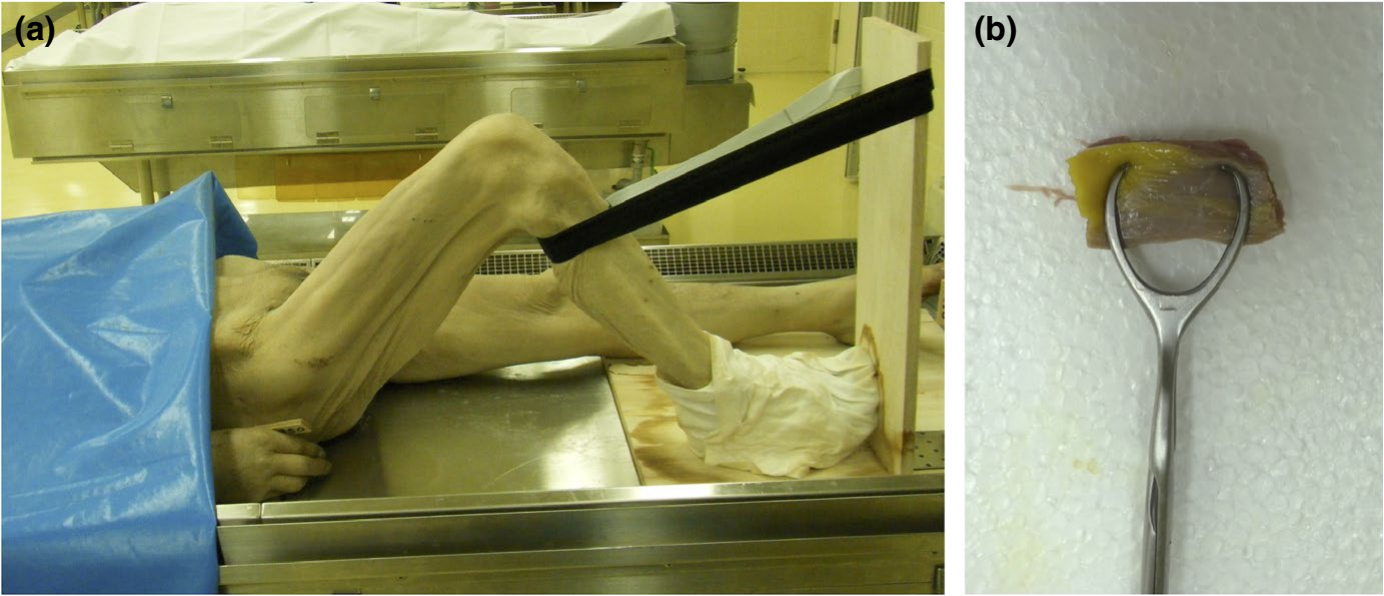
Fixed knee joint angle of 90° for the right leg (a) and muscle tissue nipped with custom‐made forceps (b).

Seven cadavers (five men and two women, age of death: 70–91 years, height: 150–168 cm, body mass: 42–67 kg) were used in this study. We excluded cadavers with knee joint contracture (e.g., with the knee joint not fully extended) and cadavers of people with reduced activity (e.g., bedridden people) before death from the study. The knee joint of the right leg was flexed and fixed at an angle of 90°, whereas that of the left leg was not treated (i.e., knee joint angle of 0°–5°) (Figure 1). Formaldehyde was used to fix the cadavers.

### 2.3 Detection of muscle tissue samples

The skin of the anterior thigh was incised, and the underlying subcutaneous tissue was removed to expose the muscles. The rectus femoris was also removed to expose the VI. After these preparations, the VL and VI were cut along each fascicle direction and perpendicular to the femur. The muscle tissues of the VL and VI were dissected at the midpoint between the greater trochanter and the inferior edge of the patella. The middle of the fascicles was carefully dissected using custom‐made forceps (Figure 1). Moo et al. (2016) indicated regional differences in the SL within a muscle. To investigate regional differences in SL in the VL and VI, muscle tissue dissections were performed at two locations in four cadavers, although the two chosen locations were not widely separated (i.e., both locations were in the middle of muscle).

### 2.4 Transmission electron microscopy (TEM) imaging

**FIGURE 2:**
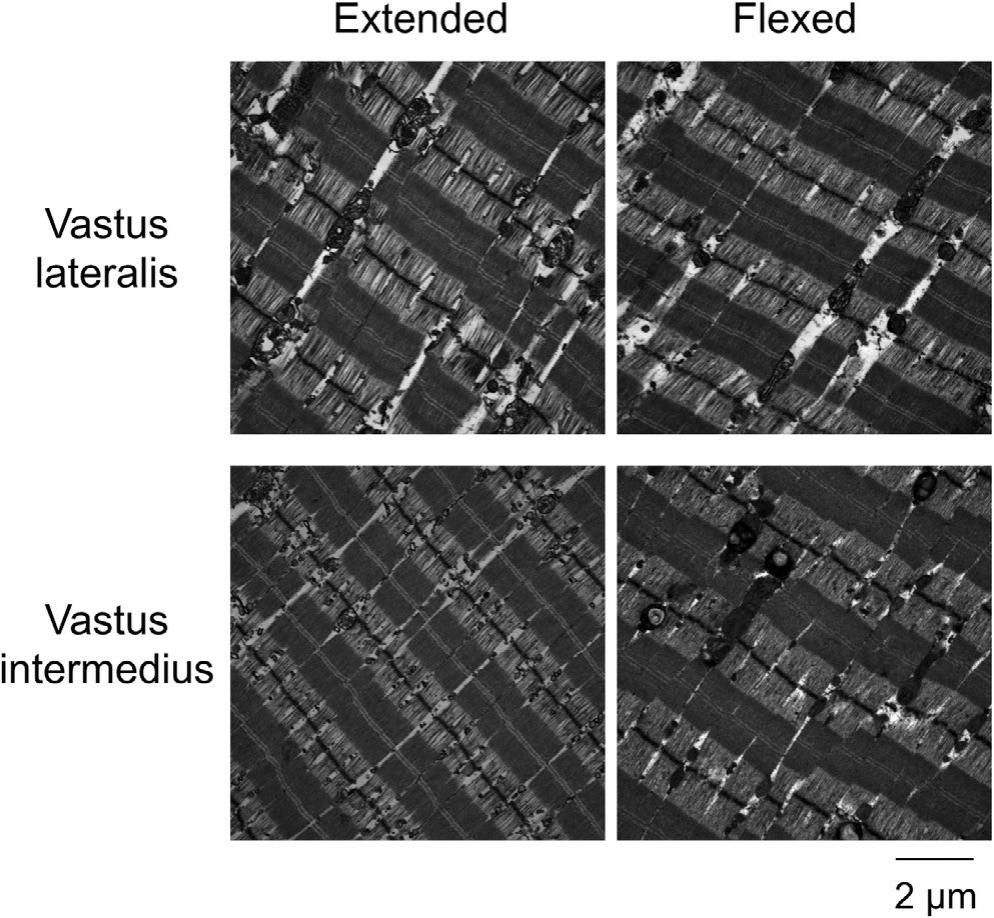
Electron microscopy image of the muscle tissues in both legs.

The procedures were almost the same as those described in a previous study (Kikuchi et al., 2018). In brief, excised muscle tissues were fixed with 4% paraformaldehyde (PFA) containing 2.5% glutaraldehyde in 0.1 M cacodylate buffer (pH 7.4) at 4°C overnight without tension‐release by custom‐made forceps, and then, ~1 mm^2^ of the sample was cropped. Next, the samples were washed with cacodylate buffer and further fixed in 1% OsO_4_ for 90 min at room temperature. The samples were then dehydrated through a graded ethanol series (60%, 70%, 80%, 90%, 99%, and 100%). They were embedded in epoxy resin, cut into sections with a thickness of 70–100 nm by using an ultramicrotome (MTX; Boeckeler Instruments), and mounted on glides. These ultrathin sections were stained with uranyl acetate and lead citrate at room temperature for 20 min and 5 min, respectively. Finally, a transmission electron microscope (JEM‐1400; JEOL Ltd.), with accelerating voltage set at 8 kV and 10 μA of beam current, was used to obtain images (Figure 2).

### 2.5 Analyses

The SL was generally defined as the length from one Z‐line to the next Z‐line (Brenda, 1983). However, muscle tissue samples might not be cut parallel to the sarcomere array, making accurate analyses impossible. Therefore, we analyzed the myosin length (A‐band length) and normalized the SL such that the myosin length was 1.6 μm (Herzog et al. 1990) as follows:

$$\text{SL}\;\left(\mu m\right)\;=\;\text{distance between Z}‐\text{lines}\times 1.6/\text{myosin length}$$

The SL has been noted to not be uniform in series within a fiber (Cromie et al., 2013; Infantolino et al., 2010). We measured the length of ten sarcomeres per image and averaged these values for use in statistical analyses. The coefficients of variation (CV) of the between‐measurement points (10 points) were 2.9 ± 1.4% and 2.4 ± 0.8% for the VL and VI, respectively, at the extended knee joint angle and 2.3 ± 0.7% and 2.7 ± 1.0%, respectively, at the flexed knee joint angle. The CVs of SL measurements at two different locations were 3.2 ± 3.0% for the VL (n = 3) and 3.0 ± 2.0% for the VI (n = 4), respectively.

Recently, SL has been measured in situ or in vivo using second harmonic generation imaging (Lichtwark et al., 2018; Moo et al., 2020). In those images, SL is measured as the distance between two adjacent M‐lines. To confirm the consistency of SL measurement between the Z‐line to Z‐line and the M‐line to M‐line, we also measured the SL by measuring the distance between M‐lines. The CV between those two analyses was 3.5 ± 3.6%, which indicates a similar distance, i.e., SL. We used SL measured from the Z‐line to Z‐line in the present study. The magnitude of change in SL with knee joint angles was calculated by subtracting the measurements at the extended knee joint angle from those at the flexed knee joint angle.

### 2.6 Statistical analyses

Statistical analyses were performed using the IBM SPSS Statistics software (version 24.0; IBM). This study used a nonparametric test because the Shapiro–Wilk test did not indicate a normal distribution. SL was compared between knee joint angles using the Wilcoxon rank sum test. The magnitude of change in SL was compared between muscles using the Mann‐Whitney U test. *r* was calculated as the index of the effect size. The values of *r* were interpreted as trivial for *r* < 0.10, small for 0.10 ≤ *r* < 0.30, medium for 0.30 ≤ *r *< 0.50, and large effects for 0.50 ≤ *r*. The level of significance was set at *p* < 0.05 for the analyses.

## 3 RESULTS

**FIGURE 3:**
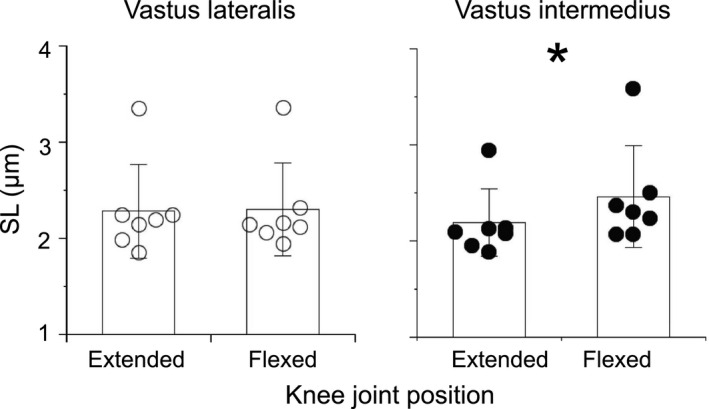
Individual and averaged values of the normalized sarcomere length (SL) for the vastus lateralis and vastus intermedius. Values are expressed as the mean ± standard deviation. SL of the vastus lateralis was not significantly different between joint angles (*p* = 0.672), whereas that of the vastus intermedius was increased at the flexed knee joint angle compared to the extended knee joint angle (*p* = 0.028). *: *p* < 0.05 significant difference between knee joint positions, Wilcoxon rank sum test.

The SL of the VL and VI is shown in Figure 3. The VL SL was not significantly different between the knee joint angles (*p* = 0.672, *r* = 0.11). The VI SL was significantly longer for the knee joint at flexion than at extension (*p* = 0.028, *r* = 0.59). The magnitude of change in SL between joint angles was significantly greater in the VI (−0.01 to 0.64 μm) than in the VL (−0.14 to 0.11 μm) (*p* = 0.009, *r* = 0.70). These statistical results remained true even when one outlier was excluded.

## 4 DISCUSSION

We indicated that the SL of the VI was longer at a knee joint angle of 90º than at that of 0º, whereas that of the VL was similar between joint angles. Furthermore, the magnitude of the SL change between joint angles was greater in the VI than in the VL. Thus, the knee joint angle changes may affect the passive and active tension produced by the VI more than those produced by the VL.

Because the SL was under 2.64 μm (optimal length of force–length curve: 2.64–2.81, (Rassier et al., 1999)) in the VI at both knee joint angles, the active SL would be on the ascending limb of the force–length curve. This assumption partly supports the previous finding by Cutts (1988) that the VI active SL changes only slightly in the ascending limb and reaches the plateau region for a knee flexion angle of 115°. Cutts’ and our studies suggest that the VI produces greater tension for large knee flexion angles. However, large knee flexion angles (>115°) are rare during daily physical activities such as walking, standing up from a sitting position, and going up or down the stairs. Therefore, the VI may not contribute significantly to these activities but rather to knee extension torque at the lowest position during full squats.

The force–length relation is determined from active SL rather than resting SL. The sarcomere shortens during muscular contraction (Moo et al., 2017). Thus, the magnitude of shortening should be considered in the calculation of active SL. There were no differences in the magnitude of VI fascicle length change during isometric muscular contraction between knee joint angles, whereas that of VL fascicle length change was greater at an extended knee joint angle compared to a flexed knee joint angle (Ando et al., 2016). Taken together, this previous finding and our results suggest that VI SL will be shorter at the extended versus the flexed knee joint angles during isometric contraction. Similarly, the VL SL will also be shorter at the extended compared to the flexed knee joint angle. However, future analysis with direct measurement is necessary to confirm these data.

The magnitude of change in SL between knee joint angles of 0° and 90° was greater in the VI than in the VL. This result may be explained by the following reasons. First, the VI has a smaller pennation angle than the VL (e.g., VI: 4.5° vs. VL: 18.4°) (Ando et al., 2016, 2018; Ward et al., 2009). From the gear ratio (Azizi & Roberts, 2014), it is assumed that the change in VI muscle–tendon unit length depends more on the change in fiber (sarcomere) length than on fiber rotation during passive lengthening, unlike the change in VL muscle–tendon unit. Second, the VI has a lower amount of tendinous tissue because the VI fibers directly attach to the femur (Ando et al., 2014). This suggests that the change in VI muscle–tendon unit length further depends on the change in fiber (sarcomere) length, although the VL muscle–tendon unit partly depends on the elongation of tendinous tissues. Finally, there was evidence for a greater moment arm in the VI than in the VL when the knee was flexed (Hume et al., 2018). Overall, the greater SL change in VI with knee joint angle changes could be attributed to architectural and anatomical features. These factors had not been fully considered when estimating the active SL at flexed knee joint angles in previous studies (Cutts, 1988; Herzog et al., 1990); by contrast, we directly measured the SL at flexed knee joint angles in the present study.

Noorkoiv et al. (2014) reported that isometric strength training at a longer muscle length, compared with that at shorter muscle length, had a greater effect on muscle volume in the quadriceps femoris. They suggested that protein synthesis was promoted by greater metabolic stress at a longer muscle length (Russ, 2008). Our results will be helpful while considering training regimens to improve strength following negative effects of aging or disuse; it will be important to consider aspects such as joint angle or where a range of motion has the most effect on muscle strength and muscle volume, although the active SL was not calculated in the present study. Because the change in SL was different for VI and VL with joint angle, muscle specific hypertrophy may be expected with resistance training at specific knee joint angles. Further study is warranted to test this hypothesis.

There were three limitations in the present study. First, all cadavers were from older adults. This implies that the relationship between the SL and knee joint angle in the present study may not reflect that in younger people because of differences in the nature of soft tissues due to age and physical activity. In particular, with aging, the tendon strain decreased (Kubo et al., 2007) and passive muscle stiffness increased linearly (Eby et al., 2015). Furthermore, with inactivity, the mechanical properties of the muscle–tendon unit changed even in younger people (Kubo et al., 2004; Reeves et al., 2005). Finally, Gokhin et al. (2014) demonstrated the change in thin actin filament length with age. These factors could be related to outlier data, as one cadaver was aged 74 years and died by hanging, suggesting that he would not have been bedridden (conversely, he was likely able to do moderate physical activity) before death, as were the other six subjects. Second, muscle tissues were dissected from the middle of the muscle. It has been indicated that the change in SL with joint angle change is different depending on measurement site, e.g., proximal, middle, or distal (Moo et al., 2016). Therefore, regional differences should be considered in the relationship between the SL and joint angle. Third, hip joint angles in cadavers in the present study were not consistent with the general posture of knee extension strength measurement on the dynamometer in vivo. Because the VL and VI are not bi‐articular muscles, it may not be necessary to take the hip joint angle into account. However, a prior study has suggested a unique VI function for different hip joint angles (Saito & Akima, 2015). Thus, we may need to account for hip joint angle in estimation of the VI active SL during dynamic multi‐joint exercises.

## 5 CONCLUSIONS

We demonstrated that the resting SL in the VI was less than 2.64 μm at knee joint angles of 0º and 90º. This result suggests that the VI SL is on the ascending limb of the force–length curve during muscular contraction. The magnitude of change in SL with knee joint angle changes was greater in the VI than in the VL, suggesting that knee joint angle changes may affect the passive and active tension produced by the VI more than those produced by the VL.

## CONFLICT OF INTEREST

The authors declare that there are no conflicts of interest.

## AUTHOR CONTRIBUTION

R.A., K.T., and H.A. conceived and designed research; R.A., K.T., S.K., S.M., and H.A. performed experiments; R.A. analyzed data; R.A., K.T., S.K., and H.A. interpreted results of experiments; R.A. prepared figures; R.A. drafted the manuscript; all authors edited and revised the manuscript; and all authors approved the final version of manuscript.

## ETHICAL STATEMENT

All procedures were approved by the Ethics Committee of the School of Medicine, Sapporo Medical University, and the Research Center of Health, Physical Fitness and Sports, Nagoya University.
